# Non-Spinal Neuromodulation of the Lumbar Medial Branch Nerve for Chronic Axial Low Back Pain: A Narrative Review

**DOI:** 10.3389/fpain.2022.835519

**Published:** 2022-02-25

**Authors:** Vinicius Tieppo Francio, Benjamin D. Westerhaus, Adam Rupp, Dawood Sayed

**Affiliations:** ^1^Department of Rehabilitation Medicine, The University of Kansas Medical Center (KUMC), Kansas City, KS, United States; ^2^Cantor Spine Center at the Paley Orthopedic and Spine Institute, Ft. Lauderdale, FL, United States; ^3^Department of Anesthesiology, The University of Kansas Medical Center (KUMC), Kansas City, KS, United States

**Keywords:** peripheral nerve stimulation, multifidus, lumbar medial branch, low back pain, neuromodulation

## Abstract

Chronic low back pain remains highly prevalent, costly, and the leading cause of disability worldwide. Symptoms are complex and treatment involves an interdisciplinary approach. Due to diverse anatomical etiologies, treatment outcomes with interventional options are highly variable. A novel approach to treating chronic axial low back pain entails the use of peripheral nerve stimulation to the lumbar medial branch nerve, and this review examines the clinical data of the two different, commercially available, non-spinal neuromodulation systems. This review provides the clinician a succinct narrative that presents up-to-date data objectively. Our review found ten clinical studies, including one report of two cases, six prospective studies, and three randomized clinical trials published to date. Currently, there are different proposed mechanisms of action to address chronic axial low back pain with different implantation techniques. Evidence suggests that peripheral nerve stimulation of the lumbar medial branch nerve may be effective in improving pain and function in patients with chronic axial low back pain symptoms at short and long term follow up, with good safety profiles. Further long-term data is needed to consider this intervention earlier in the pain treatment algorithm, but initial data are promising.

## Introduction

Axial low back pain (LBP) is a complex syndrome involving nociceptive and neuropathic pain with potential for compensatory structural changes in ligamentous and myofascial components, leading to a challenging diagnosis and therapeutic course in clinical practice (1). Chronic axial LBP is defined as pain localized along the lower back region without radicular or referred pain pattern into the extremities, lasting for at least 6 months and is often associated with complex biopsychosocial factors leading to significant impairments in function and quality of life (QoL) (1, 2). Acute LBP is a common health problem that affects roughly 75–85% of all people at some point in their life, and it is the second most common reason for physician visits (3–5). Fortunately, most cases of acute LBP have a favorable outcome, with the majority of patients fully recovering within the first 2–4 weeks, and 90% of patients recovering by 12 weeks. However, recurrence of LBP is common, with rates of up to 50% within 1 year (6). In addition, each subsequent episode connotes a 10–15% chance for chronicity (7). Owing to this high prevalence and incidence of recurrence, the economic impact is staggering, with the majority of costs deriving from missed work days, rather than direct healthcare costs. Approximately only 50% of patients who take off work for 6 months due to LBP return to work, and an estimated 1% of the U.S. population is chronically disabled because of LBP (5–8).

The etiology of axial LBP is vague and difficult to determine precisely. Estimates suggest <15% of LBP causes are identified, leaving the majority idiopathic (7, 9, 10). Some identifiable causes of axial LBP include myofascial pain, discogenic pain, facet-mediated pain, vertebral endplate injury, disc herniation without radiculopathy, central or neuroforaminal spinal stenosis without radiculopathy, sacroiliac joint pain, and fibromyalgia. Other less common causes are osteoporotic fracture, metastatic or primary neoplasm, infection, rheumatologic conditions including ankylosing spondylitis, or vascular conditions like abdominal aortic aneurysm rupture (7, 10) An overlooked etiology of chronic axial LBP is the complex interaction between multifidus muscle and morphology changes leading to dysfunction and impaired sensorimotor control of lumbar intervertebral stability (9, 11–13). Oftentimes, the diagnosis is not simply one pathology, but rather combining overlapping conditions, which suggests the multifactorial nature of axial LBP. Despite the ongoing quest to find Occam's razor for LBP, physicians work to treat the simplest cause of low back pain given a thorough history, physical exam, and imaging.

Management of axial LBP focuses on conservative therapies such as ice, topicals, antiinflammatories, light stretching, professionally directed physical therapy, and gradual return to activity. With increasing pain, disability, pain duration, or failure of treatment, interventional approaches are subsequently attempted. Common interventional modalities include epidural steroid injections (ESI), medial branch blocks (MBB), intraarticular facet injections, lumbar radiofrequency ablation (RFA) of medial branches of dorsal rami or basivertebral nerve, mechanical surgical procedures (including posterior stabilization, interspinous spacers, or minimally invasive decompression) and neuromodulation, including spinal cord stimulation (SCS), or peripheral nerve stimulation (PNS) (10, 14, 15).

This review seeks to examine the current concepts of PNS of the lumbar medial branch nerve (LMBN) of the dorsal ramus by looking at current clinical data and contrasting with other interventional treatments options for chronic axial LBP.

## Methods

This study is a review aimed at appraising current concepts in non-spinal neuromodulation for chronic axial LBP, particularly PNS of the LMBN. Data sources included PubMed, MEDLINE, Google Scholar, and Cochrane Library indexed manuscripts. Literature search was conducted between July 2021 and October 2021 for entries with keywords of “peripheral nerve stimulation” and “multifidus” and “lumbar medial branch.” Inclusion criteria were human studies in the English language, such as randomized trials, observational studies, prospective studies, and case series. All records were identified and appraised independently by the authors in a standardized, unbiased, unblinded fashion using the same approach to reduce the risk of selection bias and standardized inclusion and exclusion criteria. Book chapters, non-human clinical studies, and letters to the editors were excluded (Table 1). For all studies included, data synthesis, data analyses, quality appraisal, outcome measurements, and risk of bias assessment were performed by the authors. Data extracted from current published studies are summarized in Table 2.

**Table 1:** Inclusion and exclusion criteria of the literature review.

**Inclusion criteria**	**Exclusion criteria**
Original peer-reviewed research	Book chapters, letters to the editor, websites, etc.
English language	Manuscripts in languages other than English
Human studies	Non-human studies
Intervention specifically targeting the lumbar medial branch	Duplicate data

**Table 2:** Summary of findings.

**Author, year**	**Study type**	**Inclusion (I) and Exclusion (E)**	**Level of Evidence**	**N**	**Device and duration**	**Pain outcomes**	**Functional outcomes**	**Adverse events**
Thomson et al. (16)	P-MC Longitudinal Cohort	I: >90 days LBP, refracotry to ctherapy and medications E: surgical indication	2b	37	ReActive 2 yr	NRS BL 7.0 ± 0.2 2yr 3.5 ± 0.3 (*p* <0.001)	ODI BL 46.2 ± 2.2 2yr 29.2 ± 3.1 (*p* <0.001) EQ-5D BL 0.426 ± 0.035 2yr 0.675 ± 0.030 (*p* <0.001)	
Deer et al. (17)	P-MC	I: >12 weeks LBP with failure to >4 weeks of medications, BPI-5>4 points E: Radiular symptoms, prior lumbar surgery, spinal injections 3 months prior, lumbar RFA 6 months prior, scoliosis, BMI>40	2b	15	SPRINT 5 months	BPI-5 BL 6.3 +/- 1.0 2 mo 2.4 +/- 1.6 5 mo 3.1 +/- 1.9 BPI-9 BL 6.2 +/- 1.8 2 mo 2.4 +/- 2.1 5 mo 3.2 +/- 2.7 *p* <0.05 for all parameters	ODI BL 43.1 +/- 12.7 2 mo 21.8 +/- 13.9 5 mo 26.1+/- 13.2 *p* <0.05	4 lead migration 1 skin irritation 1 skin infection
Mitchell et al. (18)	P-MC single arm	I: chronic LBP >90 days refractory to medical management, ODI 25–60% and average NRS 6–9 E: BMI>35, surgical indication, RFA within 12 months, leg>back pain, scoliosis, morphne miliequivalent >120 mEq, spinal injections 30 days prior	2b	53	ReActiv8 4 years	NRS BL 6.8± 0.8 4 yr 3.2 ± 0.4 *P* <0.05	ODI BL44.9± 10.1 4 yr 23± 3.2 EQ-5D BL 0.434± 0.185 4 yr 0.721± 0.035 *P* <0.05 for all parameters	
Gilligan et al. (19)	DB-RCT-MC sham-control	I: mechanical CLBP >90 days refractory to emdical treatmnt, prior week average VAS 6–9, ODI 21–60 E: Prior lumbar surgery, surgial indication, leg>back pain, scoliosis, RFA within 12 months, spinal injectionswthin 30 days, morphne equivalent >120 mEq	1b	204	ReActiv8 120 days	VAS change Tx −3.3 ± 2.7 Sham −2.4 ± 2.9 95% CI −0.9 (−1.6, −0.1) *P* = 0.032	ODI change Tx −17.5 ± 15 Sham −12.2 ± 14.6 95% CI −5.4 (−9.5, −1.2) *p* = 0.01 EQ-5D change Tx 0.186 ± 0.199 Sham 0.115 ± 0.178 95% CI 0.071 (0.018, 0.123) *p* = 0.009	3.9% infection 2.9% lead replacement 4.9% revision
Gilmore et al. (20)	P-MC	I: LBP >12 weeks, failed conservative treatment E: Radicular pain, prior lumbar surgery, epidural within 3 months, RFA within 6 months, severe scoliosis, BMI ≥40, BDI-II >20	2b	74	SPRINT 14 months	BPI-5 BL 6.1 +/-1.2 14 mo 3.9 +/-202 *p* <0.05 BPI-9 BL 5.6 +/- 2.1 14 mo 3.5 +/- 2.3 *p* <0.05	ODI BL 38.5 +/-12.5 14 mo 29.5 +/- 15.3 *p* <0.05	28 skin irritation 19 itching 6 increased pain 2 infection
Gilmore et al. (21)	Prospective case series	I: LBP >12 weeks refractory to >4 week trial of medications, week average BPI >4 E: Radicular, prior lumbar surgery,	2b	6	SPRINT 12 months	BPI-5 63% reduction 95 CI (0.28, 1.04)	ODI 32 pt reduction Statistically significant	2 skin irritation
		epidural within 3 months, RFA within 6 months, BMI ≥40, BDI-II > 20						
Gilmore et al. (22)	Prospective case series	I: LBP >12 weeks refractory to >4 weeks medications, BPI week average >4 E: Radicular, prior lumbar surgery, epidural within 3 months, RFA within 6 months, BMI ≥40, BDI-II >20	2b	9	SPRINT 4 months	BPI-5 67 >50% reduction *p* <0.05	ODI 67 >10 point reduction 95%CI (0.36–0.97) *p* <0.05	2 skin irritation
Cohen et al. (23)	Case-series	I: LBP >3months refractory to >4 week trial of medications, BPI >4 E: Radicular, prior lumbar surgery, epidural within 3 months, RFA within 6 months, BDI-II >20	4b	9	SPRINT 1, 4, 7, months	BPI-5 BL 5.7 1 mo 2.2 4 mo 2.3 7 mo 4.1 *p* <0.0096 BPI-9 BL 5.1 1 mo 2.2 4 mo 2.9 7 mo 5.1	ODI BL 32.9 1 mo 18.5 4 mo 19.4 7 mo 26.1	2 skin irritation
Deckers et al. (24)	P-MC single arm trial	I: chronic LBP >90 days refractory to medical management, ODI 25%−60%, average NRS 6–9 E: BMI ≥35, surgical indication, leg > back pain, severe scoliosis, neurological deficit, severe scoliosis, morphine >120 meq, RFA in last year, MBB or epidural in last 30 days, prior lumbar surgery, depression	2b	53	ReActiv8 60, 90, 365 days	NRS change from BL 90d 2.5 ± 0.3 (*p* <0.0001) 6 mo 2.2 ± 0.4 (*p* <0.0001) 1 yr 2.4 ± 0.4 (*p* <0.0001)	ODI improvement 90d 13.4 ± 2.2 (*p* <0.0001) 6 mo 11.6 ± 2.4 (*p* <0.0001) 1 yr 14.3 ± 2.3 (*p* <0.0001) EQ-5D improvement 90d 0.213 ± 0.025 (*p* <0.0001) 6 mo 0.184 ± 0.032 (*p* <0.0001) 1 yr 0.219 ± 0.028 (*p* <0.0001)	14 increased pain 39 lead discomfort 14 undesired sensation
Kapural et al. (25)	Report of two cases	I: LBP > 3 months refractory to >4 week trial of emdications, week average BPI >4 E: Prior lumbar surgery, epidural within 3 months, RFA within 6 months, BDI-II >20	4b	2	SPRINT 4 months	BPI-5 Subject 1 BL 5.0 4 mo 1.0 79% reduction Subject 2 BL 5.0 4 mo 1.0 79% reduction	ODI Subject 1 BL 17 4 mo 18 Subject 2 BL 50 4 mo 8 87% improvement	1 skin irritation

*N, number of subjects; P, Prospective; MC, Multicenter; DB, Double Blind; BPI-5/9, Brief Pain Inventory; NRS, Numerical rating scale; VAS, visual analog scale; ODI, Oswestry Disability Index; EQ-5D, European Quality of Life Five Dimension; BL, Baseline; BDI, Bec Depression Inventory; Tx, treatment; RFA, radiofrequency ablation; MBB, medial branch block*.

## Results

Currently, there are two types of PNS implantation systems targeting the LMBN: the 60-day percutaneous PNS implant (SPRINT) and the permanently implanted restorative LMBN PNS (ReActiv8). The SPRINT platform proposes a mechanism that is palliative and theorized to modulate afferent pain signals that originate peripherally and move centrally, by means of a fine wire electrode. The ReActiv8 system proposes a mechanism that is restorative and theorized to stimulate efferent signals that originate with the device and cause the multifidus muscle to contract in a rehabilitative fashion. Our search found 25 results for peripheral nerve stimulation and multifidus and 23 results for peripheral nerve stimulation and lumbar medial branch nerve keywords. Forty-eight results were accessed for eligibility, 38 results were excluded from Table 1 since these did not involve human clinical data and/or were duplicates. A total of 10 clinical studies were included, including one randomized controlled trial, one report of two cases and eight prospective case series. These were reviewed, appraised, summarized, and listed in Table 2.

## Discussion

Chronic axial LBP is a highly prevalent and debilitating diagnosis associated with complex biopsychosocial factors treated with inconsistent management and limited successful outcome rates due to numerous anatomical etiologies (10, 26). Due to its complexity, it often requires an interdisciplinary rehabilitative treatment with numerous interventional procedures available for targeting chronic axial LBP, such as epidural steroid injections, facet joint injections, nerve blocks, SCS, etc. each with varying levels of efficacy given the poor specificity of the diagnosis.

There has been a lack of well-designed randomized, controlled studies to determine the effectiveness of epidural steroid injections (ESI) for chronic axial LBP alone, though evidence may favor its use in radicular LBP. Abdi et al. (27) found the evidence is indeterminate in the management of axial LBP and Staal et al. (28) concluded that there was no strong evidence for or against the use of ESI for chronic axial LBP (27, 28). More recently, Manchikanti et al. (29, 30) systematic review and randomized clinical trial found fair evidence for the utilization of caudal and interlaminar ESI and limited for transforaminal ESI (29, 30). Perhaps due to relative inexpensiveness and easy accessibility, ESI are commonly performed to manage symptoms associated with chronic axial LBP, however this targets an acute inflammatory etiology, often not the cause of more chronic axial LBP. SCS effectiveness has been well-documented for chronic lumbar radiculopathy after failed lumbar spine surgery and for complex regional pain syndrome, however there is low-quality evidence that SCS is effective in patients with axial LBP alone (26, 31–36). There are numerous proposed mechanisms of action for SCS, from dorsal column inhibition, to modulating wide-dynamic range neurons, glial cells, ascending and descending pathways; however, all these have a palliative neuromodulatory therapeutic paradigm (31, 36).

Data is slightly more favorable toward the use of intraarticular facet steroid injection for temporary palliation of facet-mediated pain. The systematic review of Manchikanti et al. (37) concluded that there is low-grade evidence for long-term improvement of facet joint pain with intraarticular facet injection, however Mayer et al. (38) found no significant difference in pain and disability comparing facet injections and exercise therapy groups, both with statistically significant improvement (37, 38). Facet joint nerve blocks of the LMBN have been shown to be of diagnostic value for facet-mediated pain, particularly prior to LMBN radiofrequency ablation (RFA). Both facet joint intraarticular injections and LMBN blocks have been shown to improve pain and function short-term, but results are equivocal at long-term (39, 40). Lumbar RFA has been shown to improve pain, function, and QoL at 6-months; however results were not significantly different when compared to intraarticular facet injections (41). There is moderate level I evidence supporting the use of LMBN RFA as a palliative interventional treatment for chronic facet-mediated pain in selected patients (42, 43). Yet, LMBN RFA may have deleterious effects, particularly from the dual innervation of the dorsal ramus of the LMBN to the facet joint and toward the multifidus muscle. LMBN RFA leads to the subsequent denervation of the multifidus muscle. Multifidus denervation leading to disuse atrophy may cause a vicious cycle of dysfunction among the three spinal stabilizing systems (spinal column, spinal muscles, and neural control unit) causing repetitive tissue injury, suboptimal stabilization of forces, improper tissue overloading, intersegmental spinal instability, impaired sensorimotor control, and chronic nociceptive and neuroplastic changes. Thus, a proposed more conservative multifidus sparing RFA would be preferred targeting the terminal end branches of the medial branch of the dorsal root, directly over the facet joint capsule (44). Targeting the LMBN has long proven to have therapeutic value. The most effective means to do so, however, has taken on several different appearances over the past decades in the interventional spine realm, from facet joints injections and blocks to neurotomy and rhizotomy.

More recently, interventional techniques have involved electrically modulating the LMBN, by either modulating pain signals from it or stimulating signals to it to induce multifidus muscle contraction. Two platforms have formally studied targeting the LMBN with differing techniques and mechanisms: SPRINT and ReActiv8. SPRINT works as a 60-day percutaneous PNS platform targeting the LMBN, utilizing a temporary device with cyclical stimulation for 6 h per day for 60 days. The SPRINT has a fine wire that can be implanted under fluoroscopy or ultrasound at any of the LMBN unilaterally or bilaterally. At the 2-month mark, the physician removes the device, as per Food and Drug Administration (FDA) approval. Electrical parameters used by this platform were studied at a frequency of 12 Hz and an amplitude of 5–20 mA (21). Like traditional PNS, the proposed mechanism of action is primarily modulation of the afferent pain signal and disruption of the central sensitization of pain. Secondarily, there is stimulation of efferent nerve signals to activate muscles (21). The ReActiv8 device shares a similar anatomical target, the LMBN, however differs in some key elements. It is a permanent implant with a subcutaneous pulse generator utilizing a more traditional lead design with four electrodes and specialty tines to secure the leads within the intertransversarii muscle next to the L2 LMBN above the L3 transverse process bilaterally. Electrical parameters are similar to SPRINT, with frequency of 20 Hz and amplitude of about 2.5 mA; however, it has a ramp up period, then plateaus, followed by a ramp down period, giving it a more prescribed pattern for each of its two 30-min sessions per day. This is implanted under fluoroscopy, and although the device is not temporary by design, it can be explanted at patient request. The proposed mechanism of action for ReActiv8 is restorative neurostimulation with its primary effector as the efferent nerve to stimulate multifidus muscle contraction in a rehabilitative fashion to increase spinal segmental stability. Central desensitization is thought to be secondary effects of its mechanism of action (19). Similarities and differences of these platforms are summarized in Table 3.

**Table 3:** Similarities and differences between two different PNS systems for chronic low back pain.

	**SPRINT**	**ReActiv8**
Proposed mechanism of action	Palliative neuromodulation	Restorative neurostimulation
Nerve targeted	Lumbar medial branch L1–L5	Lumbar medial branch of L2
Primary effector targeted	Afferent, sensory nerve	Efferent, motor nerve
Patient selection	Chronic axial low back pain	Multifidus dysfunction evidenced by diagnostic imaging findings with multifidus muscle atrophy and/or positive physical exam maneuvers (prone instability test and multifidus lift test)
Lead wires	Fine wire electrode	Traditional lead with 4 electrode arrangement
Pulse generator	External	Subcutaneous
Imaging modality of placement	Fluoroscopy or ultrasound	Fluoroscopy
Stimulation	Low frequency, high amplitude, 6 hours per day	Low frequency, high amplitude, two 30-min sessions per day
Duration of therapy	60 days, temporary	Permanent implant

Percutaneous PNS of the LMBN is a relatively new modality, and to date, there are 10 human, clinical studies focusing on non-spinal neuromodulation for chronic axial LBP (16–25). Kapural et al. (25) was the first to publish clinical data using the SPRINT device. Two patients were included, which can only demonstrate general feasibility of the device, and clinical outcomes cannot be reasonably generalized from these two case reports. Cohen et al. (23) conducted a small prospective case series with nine subjects evaluated monthly for a year. They found a statistically significant reduction in both pain and functional scores at all time points. In addition, 83% reported improvement in their quality of life [PGIC 95% CI (0.54–1.13)], which is not a significant value (23). The low patient number is a substantial caveat to generalizing these data, as the risk of spontaneous regression is high.

Gilmore and colleagues produced much of the subsequent literature on the 60-day percutaneous PNS platform. In the 2019 study, they performed a prospective case series with nine patients over 4 months, and demonstrated statistically significant improvement in pain and function when considering >50% pain reduction and >10 point reduction on ODI, respectively (BPI-5 67 >50% reduction *p* > 0.05, ODI 67% >10 point reduction, 95%CI 0.36–0.97), *p* >0.05) (22). In this study, the reader should bear in mind the small patient sample and relative data analysis, as objective data would be more robust for extrapolating conclusions. In the 2020 study, Gilmore et al. (21) followed up on six of the previously reported patients at 12 months. This study sought to demonstrate durability of the therapy. There was a statistically significant improvement in function with 32 point reduction as measured by ODI, and there was 63% reduction in pain on BPI-5 that was not statistically significant [95 CI (0.28, 1.04)] (21). Again, with a small sample size, this study can only reasonably conclude the feasibility of the therapy.

Deer et al. (17) conducted a slightly larger prospective multicenter study with 15 subjects and extended their data collection to 5 months. The endpoints used were brief pain inventory (BPI-5), Oswestry disability index (ODI) and brief pain inventory (BPI-9). They found statistically significant pain and functional score improvements at both 2 and 5 month time periods. In addition to this 67 and 80% of patients at 2 and 5 months, respectively had >50% pain reduction. They reported similar trends in both pain interference and disability. At 2 months, 87% of the patients had statistically significant changes in two or more endpoints, which decreased to 73% at 5 months. 93% had clinically significant improvement in at least 1 category at all time points (17). The most recent and robust study of the 60-day percutaneous PNS technique comes from Gilmore et al. (20). In this study, 74 patients across multiple centers were followed for 8 months, with longer follow up visits completed in 51 patients. Pain intensity measured by BPI-5 was improved at 2, 5, and 8 months after implantation in a significant manner (baseline 6.1 to 3.3, 3.6, 3.9 at 2, 5, and 8 months, respectively, *p* < 0.05). Functional data also demonstrated significant improvement at these intervals (baseline 38.5 on ODI to 22.9, 25.0, and 27.5 at 2, 5, and 8 months, respectively, *p* < 0.05). The most common adverse events were dermatologic, with skin irritation in 34 of 89 patients (19).

The permanently implanted restorative LMBN PNS system comprises the other arm of the non-spinal neuromodulation for chronic axial LBP scheme. This device is implanted in a minimally invasive fashion, and then stimulates the medial branch of the bilateral L2 LMBN at 20 Hz for 10 s with 20 s off for 30 min twice daily. Deckers et al. (24) initiated the first investigation with 53 subjects in a prospective multicenter trial. Parameters included numeric rating scale (NRS), ODI and quality of life questionnaire (EQ-5D) measured at 3, 6 and 12 months. They found statistically significant improvements in all categories at each time point (*p* < 0.0001). Deeper analysis of the data to include minimal clinically important difference (MCID) showed that for NRS which required >2pt improvement, 63%, 61%, and 57% of patients reached MCID at 3, 6, and 12 months, respectively (*p* < 0.0001). This trend was similar to EQ-5D (MCID >0.3). ODI with a MCID >10, had an increasing trend with 52, 57, 60% patient's reaching MCID at 3, 6, 12 months, respectively. 87% of patients at 1 year had MCID in at least 1 category (24).

Following this study, Gilligan et al. (19) published the first double-blinded, randomized, multicenter control trial with 204 patients. They blinded patients and physicians by implanting all subjects and randomizing whether a patient received either therapeutic stimulation or sham stimulation. They evaluated VAS, ODI, and EQ-5D for 120 days. The primary endpoint was based on responder obtaining 30% relief on the LBP visual analog scale without analgesics increase. Then, at 120 days from implantation, the subjects in the sham group crossed over to therapeutic stimulation, and outcome measures were tabulated out to 1 year. At 120 days post-op, the results were inconclusive with a responder rate of 57.1% (treatment) vs 46.6% (sham) (difference 10.4%, 95% CI −3.3 to 24.1%, *p* = 0.138). However, deeper analysis of the data shows that though the primary outcome was not significant, the actual values of functional outcome measures were significantly improved at 120 days for the treatment group compared to the sham in ODI (difference −5.4 95% CI −9.5 to −1.2, *p* = 0.011) and EQ-5D (Difference 0.071 95% CI 0.018 to 0.123, *p* = 0.009) respectively, though VAS trended toward significance (Difference −0.9, 95% CI −1.6 to −0.1, *p* = 0.032) (19). Next, Thomson et al. (16) investigated NRS, ODI, and EQ-5D changes on 37 patients for 2 years. They found that at 2 years all outcome measures had significantly improved from baseline (*p* < 0.001) (16). This study design was a prospective, multicenter, longitudinal cohort, and although it is not level 1 evidence, it addresses most causes of bias and presents long-term data, but in a rather small sample size. An important critique is that the data analysis did not include patients who explanted due to inefficacy of the therapy, so the true responder rates may be over-stated. Lastly, Mitchell et al. (18) studied 53 patients in a prospective, multicenter, single arm trial that extended to4 years. Again, all outcome (NRS, ODI, EQ-5D) measures were significantly increased from baseline at the 4-year mark (*p* < 0.05). Moreover, Mitchell found that 62.5% of participants had clinically meaningful improvement in both NRS and ODI (18). In this study, though the sample size was modest, the long-term follow up is notable as it is the only study in this review with this follow up length. Again, though, true responder rate may be overstated due to the number of patients lost to follow up or explanted, which would be a helpful analysis that is not described in the paper. Overall, the complication rate with this intervention was exceedingly low in comparison with other devices, though adverse events include skin irritation and infections (21).

Both approaches of PNS of the LMBN present unique and promising approaches to treat chronic axial LBP with non-spinal neuromodulation in selected patients who are recalcitrant to conservative treatments. These provide exciting therapies to physicians whose only options previously were epidural injections, medial branch blocks, or RFA, which now may seem counter-intuitive to the therapies reviewed here. Although the two interventions have similar anatomical targets, their approaches, stimulation patterns, chronicity of treatment, and even lead design are quite different. The 60-day percutaneous PNS seeks to provide more palliative peripheral nerve stimulation of the medial branch with thin wire leads and external pulse generator, temporarily implanted for 60-days. Low-frequency pulse train stimulation of the LMBN terminal branches activates both sensory afferents and muscle efferent fibers, producing proprioceptive signals in large diameter fibers that convergently directly activate sensory afferents engaging in the spinal cord gating mechanism. Data are limited but have demonstrated sustained results, even after cessation of 60-day temporary stimulation for patients with multifactorial chronic axial low back pain, including degenerative disc disease, lumbar spondylosis, lumbar facet arthropathy, discogneic pain, etc (17, 20–23, 25). The permanently implanted restorative LMBN PNS system, on the other hand, has a more regimented minimally invasive technique. It specifically targets the L2 LMBN as it traverses over the L3 transverse process and seeks to address multifidus dysfunction to enhance sensorimotor control by restorative neurostimulation for 30 min twice a day at a higher amplitude, without expectation of device explanation. This restorative stimulation has a longer time to maximum impact, and it may take several months for a meaningful clinical improvement to accrue as it is directed to address multifidus muscle dysfunction and impaired sensorimotor control, postulated the root cause of axial low back pain with neuroplastic changes leding to chronicity (19). As research continues to grow examining these two systems, we will gain a deeper understanding of the ideal patient population best suited for each system. For now, it may be hypothesized that SPRINT may be better for the patient who has more of a neuropathic component to multifactorial chronic LBP with disc degeneration, facet arthrpathy, or seeking more immediate analgesia than restoration, without multifidus atrophy, is not able to undergo general anesthesia or otherwise does not want a permanent implant. The ReActiv8 system may be better suited for more active patients with multifidus atrophy on imaging and multifidus dysfunction on physical exam, who are seeking restorative neurostimulation and are motivated and open to the prospect of a permanent implantable device with a long-term durability effect. There is the possibility, too, that these two platforms exist on a continuum or are complementary, and both of these systems may be attempted in select patients. These two approaches can present obvious benefits and drawbacks in different patient populations, which is outside the scope of this review to discuss.

With the firm understanding in differing approaches and proposed mechanisms of action, we caveat some of this discussion with the fact that comparing two different therapies is difficult. Studies examining the permanently implanted restorative LMBN PNS system present more robust clinical data, with respect to rigor of study design, sample size, and follow up time. Their rates of patients who followed up is also slightly better, though both therapies have rather high lost-to-follow-up rates. This may be from device complication, explanation, or patients who are doing well. We also recognize that the global COVID-19 pandemic may have complicated consistent follow up as well. Yet, studies that looked at permanently implanted restorative LMBN PNS need more transparency into loss of follow up and explanation. Comparing primary endpoints of pain reduction and functional improvement, the 60-day percutaneous PNS system demonstrated better early outcomes, though durability appears to favor the permanently implanted restorative LMBN PNS system. This is an important observation considering the two different mechanisms that these therapies utilize. Neither system has examined post-procedural structural changes in the neuromuscular anatomy at long term. Further studies are warranted to investigate post-procedural imaging or electromyographic changes, and when one technology may be preferred or indicated over the other. In addition, comparing each LMBN PNS therapy vs. conventional treatment, namely LMBN blocks and/or RFA, would be an important addition to the literature. Ultimately, it would seem these two modalities—treating the nerve and destroying the nerve—are diametrically opposed. It is therefore of upmost importance for physicians to understand different anatomical etiologies of chronic axial LBP (i.e., facet-mediated pain vs. multifidus dysfunction), their therapeutic paradigms, and the proper patient selection criteria for these contrasting interventions.

As per the constraints of our review, there are only 10 studies published to date, one randomized controlled trial, one report of two cases and eight prospective studies. The available literature on PNS of the LMBN has limitations. All published studies to date have been industry sponsored, which evidently potentialize the risk of publication bias and conflict of interest. In fact, our study is non-sponsored by industry and we aimed to provide a non-biased comprehensive overview of current data on LMBN PNS of the two commercially available systems. It is always prudent to comment on the limitations of generalizability in such a setting. Data is in its infancy and more robust and larger studies are needed, including regression analysis of non-responders, subgroup analysis, and categorizing patient selection more specifically, including validation of multifidus dysfunction and standardization of inclusion and exclusion criteria in relationship to the pain generator addressed by the intervention. Perhaps this is the essential difference to clarify between these available devices, that although both target the LMBN, one addresses proposed dysfunction of the multifidus, while the other focuses on chronic axial LBP with different etiologies. Therefore, because our review evaluated different devices with proposed different mechanisms of action and clinical indications, thus a high level of heterogeneity is introduced. As we seek more personalized medicine within interventional spine and pain, we will need to continue to answer these questions to provide our patients with safe and superior outcomes.

## Conclusion

This review examines the clinical data of two different non-spinal neuromodulation systems for the treatment of chronic axial LBP. This provides the clinician a succinct narrative that presents these data objectively. Evidence suggests that PNS of the LMBN is safe and effective to improve pain and function in patients with chronic axial LBP symptoms at short and long term. With continued advancements in neuromodulation technology from ongoing research, miniaturization of devices, sophistication of procedural techniques, and wireless capability, we have the promising ability to target a highly prevalent and costly condition with PNS of the LMBN.

## Author Contributions

All authors listed have made a substantial, direct, and intellectual contribution to the work and approved it for publication.

## Conflict of Interest

DS is a research investigator for Mainstay Medical. The remaining authors declare that the research was conducted in the absence of any commercial or financial relationships that could be construed as a potential conflict of interest.

## Publisher's Note

All claims expressed in this article are solely those of the authors and do not necessarily represent those of their affiliated organizations, or those of the publisher, the editors and the reviewers. Any product that may be evaluated in this article, or claim that may be made by its manufacturer, is not guaranteed or endorsed by the publisher.
